# Pitolisant-associated mania – a case report

**DOI:** 10.1186/s12883-026-04661-7

**Published:** 2026-01-29

**Authors:** Maximilian Zoltek, Richard Ågren

**Affiliations:** Department of Physiology and Pharmacology, Karolinska Institutet, Stockholm, Sweden

**Keywords:** Pitolisant, Mania, Narcolepsy, Electroconvulsive therapy, Case report

## Abstract

**Background:**

Narcolepsy is a chronic neurological disorder treated with medications that promote wakefulness. Pitolisant, a histamine H3 receptor inverse agonist, is used to reduce excessive daytime sleepiness. Although pitolisant clinical trials describe mainly mild adverse effects, severe psychiatric reactions have been reported, yet detailed clinical descriptions remain limited. This case describes a first-onset manic episode with psychotic features shortly after pitolisant initiation in an adult without any psychiatric history, highlighting a rare but clinically significant potential reaction.

**Case presentation:**

A 51-year-old woman newly diagnosed with narcolepsy type 2 without psychiatric history began treatment with pitolisant, titrated up to 36 mg daily. She did not use recreational substances and had hypothyroidism well-managed with levothyroxine. After swift initial improvement in wakefulness, she developed behavioral changes including heightened sociability, impulsivity, and decreased need for sleep. Within weeks, the condition progressed to a severe manic state characterized by paranoid ideation, auditory hallucinations, and disinhibition. Primary care treatment with low-dose sedatives demonstrated minimal effect, resulting in referral for involuntary psychiatric admission. Comprehensive neurological, infectious, metabolic, and autoimmune evaluations were without remarks. Pitolisant was discontinued upon admission. Management included high-dose olanzapine, lorazepam, and subsequently electroconvulsive therapy, after which the manic episode resolved. She was stabilized on risperidone and olanzapine, which were gradually tapered as her condition improved. At follow-up two months after discharge, she remained euthymic and was referred to primary care.

**Conclusions:**

This case demonstrates a severe manic episode with psychotic symptoms emerging shortly after pitolisant initiation in an adult without prior psychiatric vulnerability. The temporal association, absence of alternative medical explanations, and resolution after drug discontinuation suggest a possible medication-related trigger. Clinicians prescribing pitolisant should closely monitor for early behavioral or mood changes, particularly during dose increases, and recognize that significant psychiatric reactions may occur even in individuals with no prior mental health history.

## Background

Narcolepsy is a chronic, lifelong neurological disorder characterized by excessive daytime sleepiness, cataplexy, hypnagogic or hypnopompic hallucinations, sleep paralysis, and fragmented sleep [1, 2]. Cataplexy refers to a sudden, temporary loss of muscle tone that is typically triggered by strong emotions [3, 4]. Symptoms typically emerge during adolescence [5] and persist throughout life, affecting approximately 50 per 100,000 individuals and significantly impairing quality of life [1, 2, 5–8]. Narcolepsy is classified into two subtypes; cataplexy is a defining diagnostic feature of narcolepsy type 1, but is absent in narcolepsy type 2 [9, 10]. Narcolepsy type 1 is thought to result from the loss of hypocretin producing neurons in the hypothalamus, whereas these neurons remain largely intact in narcolepsy type 2 [9–12]. In certain cases, reduced hypocretin levels may result from brain injury; however, the exact underlying mechanisms remain unknown in most instances [1, 13]. Narcolepsy type 2 is thought to result from a multifactorial combination of genetic predisposition, environmental exposures, and possibly autoimmune mechanisms.

Pharmacological management of excessive daytime sleepiness in narcolepsy may involve single or combination therapies, which are broadly categorized as stimulants or non-stimulants. The adverse effects of stimulants are well documented and include both psychiatric symptoms (e.g., insomnia and decreased appetite) and somatic effects, primarily cardiovascular in nature [14, 15]. Furthermore, clinical treatment with amphetamines is linked toan increased risk of hospitalization for mania or psychosis, whereas methylphenidate does not show the same association [16, 17] and modafinil-related psychosis has only been reported in a single case [18]. In contrast to stimulants, non-stimulants form a highly heterogeneous group. Among them, pitolisant, a novel agent, is widely recommended as a first-line therapy for excessive daytime sleepiness in both narcolepsy type 1 and 2 (marketed as Wakix^®^; approved by the European Medicines Agency in 2016 and by the Food and Drug Administration in 2019) [2, 19].

Pitolisant acts by affecting histaminergic signaling. Histamine neurons in the tuberomammillary nucleus of the hypothalamus project throughout the prefrontal arousal circuits and are selectively active during wakefulness [20]. The drug promotes wakefulness by acting as a histamine H3 receptor inverse agonist, thereby blocking histaminergic autoregulation and enhancing histamine H1 receptor signaling. Additionally, through histamine H3 heteroreceptor activity, pitolisant stimulates release of other wakefulness-related neurotransmitters, such as noradrenaline and dopamine [21]. Pitolisant is administered orally once daily at doses up to 36 mg, displays minimal abuse potential, where the most frequent adverse effects encompass headache, insomnia, and anxiety. With this profile, pitolisant is a widely used treatment option for adults with narcolepsy [2, 9, 10, 19, 22]. However, further post drug-approval studies are needed to comprehend rare adverse events. This study describes a case of psychiatric hospitalization for a manic episode with psychotic features in a patient with narcolepsy type 2 treated with pitolisant.

## Case presentation

### Patient demographics

A 51-year-old Caucasian woman, residing with her husband and adolescent child, employed in a daytime office role without shift work. She is a non-smoker, does not use recreational substances, and reports low and sporadic alcohol consumption.

### Medical history

**Fig. 1:**
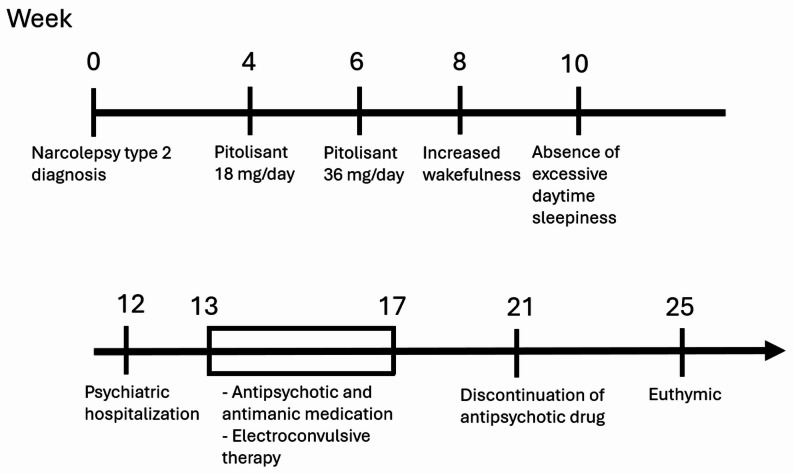
Timeline depicting the clinical course in weeks from diagnosis to curation. The electroconvulsive therapy is shown as a rectangle

The patient had no prior psychiatric history, no suicide or self-harm attempts, and no family history of bipolar or psychotic disorders. Her medical history is notable for hypothyroidism, adequately managed with levothyroxine (100 µg three times weekly and 50 µg four times weekly). She was diagnosed by a neurologist with narcolepsy type 2 twelve weeks prior to hospitalization, presenting primarily with excessive daytime sleepiness manifesting as daily midday naps, without a history of cataplexy. Eight weeks prior to hospitalization, she was prescribed pitolisant 18 mg once daily, which after two weeks was increased to 36 mg daily (Fig. 1). Methylphenidate was offered but declined by the patient.

### Presentation and symptom course

Family members reported initial benefits from pitolisant, including increased wakefulness and energy, although midday naps persisted. Approximately two weeks prior to psychiatric hospitalization, midday naps ceased, and the family noted onset of unusual and impulsive behaviors, including excessive social interaction and spontaneous job applications. In the days immediately preceding hospitalization, her condition rapidly deteriorated with development of psychotic features, including paranoid ideation, auditory hallucinations, stereotyped and irrational behaviors, megalomaniac self-perception, and disinhibition. The family reported nightly wandering and writing of a 30-page manifest. No concurrent somatic symptoms (e.g., seizures or signs of infection) were noted. Her general practitioner prescribed low-dose oxazepam and quetiapine with minimal effect one day prior to admission to a closed psychiatric ward.

### Intervention and treatment

On admission, pitolisant was suspected to be associated with the symptomatology and discontinued. Comprehensive diagnostic evaluations, including neurological examinations were without remarks. Magnetic resonance imaging with and without contrast revealed no intracranial process, hemorrhage or ischemia. Electroencephalography did not indicate epileptiform activity. The electrocardiogram was without remarks. Extensive blood analyses were without remarks except for slightly elevated plasma cholesterol. Cerebrospinal fluid analyses demonstrated no signs of hemorrhage or neuroinflammation and excluded autoimmune encephalitis.

Psychiatric management included olanzapine, titrated up to 30 mg/day, and lorazepam (up to 4 mg/day). Due to increasing confusion, insomnia, and poor nutritional intake, a decision was made to offer electroconvulsive therapy on the fifth day of hospitalization. After three treatment sessions the condition improved and the patient was able to follow conversations with only a slight forcefulness. In total, a series of ten bitemporal treatments were administered. Concurrently, risperidone (3 mg/day) and as-needed olanzapine (10 mg) were administered. By the fifth week, her condition had stabilized, permitting discharge with gradual tapering and discontinuation of antipsychotics as clinically indicated.

### Follow-up

After hospital discharge, the patient was monitored by a specialized bipolar disorder unit. Pitolisant treatment was not reinitiated. One month after hospital discharge, risperidone treatment was gradually tapered and discontinued, and olanzapine 5 mg was used per need. At follow-up two months after hospital discharge, she remained euthymic and was referred to her general practitioner.

## Discussion

We report a case of a severe first-time manic episode with psychotic features presenting shortly after initiation of treatment with pitolisant; a pharmacological agent with a novel mechanism of action. Notably, 6 possible cases of mania associated with pitolisant have been reported in a post-marketing review of over 5 million reported adverse events [23]. 

The development of a manic state approximately 6 weeks after initiation of pitolisant suggests a temporal correlation. There were no established risk factors of mania, such as a history of prior psychiatric disorders, substance abuse, or treatment with other medications known to disrupt sleep. Clinical examinations and investigations excluded a somatic or drug-induced etiology, including autoimmune encephalitis, e.g., anti-N-methyl-D-aspartate receptor encephalitis that may present with mania and at the patients’ age at diagnosis (51 years) [24–26]. Although the psychiatric presentation could theoretically represent the first episode of bipolar disorder type 1 or schizoaffective disorder, the patient’s age at diagnosis and lack of prior psychiatric history reduce the likelihood. Likewise, extreme psychological stress, which can precipitate mania, was not present. Conclusively, no obvious alternative explanation for the mania, other than pitolisant treatment, could be identified. Although rechallenge with pitolisant was not attempted, no recurrence of hypomania or mania was observed during follow-up when discontinuing the antipsychotic treatment. Applying the Naranjo scale for characterizing the likelihood of a drug adverse effect [27], pitolisant is considered a possible cause of the reported mania.

Mechanistically, pitolisant is a histamine H3 receptor inverse agonist and its wake-promoting effects are thought to be mediated via histamine H1 receptor stimulation and indirectly by increasing the release of key neurotransmitters, including dopamine and noradrenaline, through heteroreceptor activity [22]. In comparison, traditional stimulants used for attention-deficit hyperactivity disorder, such as amphetamines, act primarily by directly releasing dopamine or inhibiting its reuptake, with mania documented as a possible adverse effect. Although pitolisant is not classified as a stimulant, its ability to indirectly modulate dopaminergic and noradrenergic signaling may contribute to sustained wakefulness and, potentially, the development of mania with psychotic features.

While pitolisant is generally well tolerated, this case highlights the importance of careful monitoring for emergent psychiatric symptoms, particularly during dose escalation. Clinicians should remain vigilant for signs of mania or psychosis, even in patients without prior psychiatric history.

## Patient perspective

The patient reported insight into her condition and was appreciative of the care provided at the time of dispatchment from the psychiatric unit.

## Data Availability

Data sharing is not applicable to this article as no datasets were generated or analysed during the current study.
